# Screening of sperm antigen epitopes by phage display technique and its preliminary clinical application

**DOI:** 10.1186/s12610-022-00172-w

**Published:** 2022-11-17

**Authors:** Jin-Chun Lu, Yan-Mei Ge, Yuan-Hua Xu, Shan-Shan Tang, Yuan-Jiao Liang

**Affiliations:** Center for Reproductive Medicine, Zhongda Hospital, Southeast University, 3 Xinmofan Road, Jiangsu, 210037 China

**Keywords:** Phage display technique, Sperm antigen, Mimotope, Immunological infertility, Antisperm antibody, Standardization, Technique « phage display», Antigène spermatique, Mimotope, Infertilité immunologique, Anticorps anti-spermatozoïdes, Standardisation

## Abstract

**Background:**

At present, there is a lack of standardized preparation methods of sperm antigen for the detection of antisperm antibody (AsAb). To screen sperm antigen mimotopes from a phage display random peptide library and use them to establish an enzyme-linked immunosorbent assay (ELISA) for the detection of AsAb, immunoglobulins were extracted from the sera of rabbits with positive AsAb and negative AsAb, respectively, by the saturated ammonium sulfate method, and a phage display 12-mer peptide library was affinity panned by the extracted immunoglobins coated on the ELISA plate. Then, the obtained positive phage clones were identified by ELISA and sent for sequencing and peptides synthesis. Last, a diagnostic ELISA was established to detect clinical serum and seminal plasma samples.

**Results:**

A total of sixty phage clones were chosen by affinity panning, and sixteen of them reacted positively with AsAb in indirect ELISA and sandwich ELISA. Following DNA sequencing and translation, the peptide sequences of the sixteen positive clones were obtained. By comparison in Blast database, four of sixteen positive clones were found to be closely related to male reproduction. Two (#1 and #25) of four mimotopes were synthesized, and an ELISA method was established using the two mimotopes as sperm specific antigens. One hundred and thirty-four serum samples and seventy-four seminal plasma samples from infertile couples were analyzed by the established ELISA with #1 and #25 mimotopes, respectively. The positive rates of AsAb in serum samples were 20.15% (27/134) for #1 and 11.19% (15/134) for #25, respectively, and the coincidence rate between them was 91.04% (122/134). The positive rates of AsAb in seminal plasma samples were 1.35% (1/74) for both #1 and #25, and the coincidence rate was 100%.

**Conclusion:**

Sperm antigen mimotopes can be obtained successfully by the phage display technique, and can be used as standard sperm specific antigens to establish an ELISA method for the detection of AsAb.

## Résumé

**Contexte:**

À ce jour, il n'existe pas de méthodes normalisées de préparation d'antigènes spermatiques pour la détection des anticorps anti-spermatozoïdes (ACAS). Dans le but d’élaborer un tel test ELISA (enzyme-linked immunosorbent assay), nous avons extrait de sérum de lapins des anticorps anti-spermatozoïdes humains via la technique du sulfate d’ammonium saturé et en ayant recours à une librairie phagique de peptides (12-mer). Les clones positifs ont été identifiés par ELISA, séquencés à façon et les peptides correspondants ont été synthétisés. *In fine*, un test ELISA diagnostic a été conçu pour être utilisé avec des échantillons cliniques de sérum et de plasmas séminaux.

**Résultats:**

Au total, soixante clones de phages ont été sélectionnés, et seize d’entre eux se sont avérés interagir avec les ACAS en ELISA indirect comme en ELISA sandwich. Les séquences peptidiques de ces seize clones positifs ont été obtenues. Par comparaison avec les bases de données (Blast), quatre de ces seize clones positifs se sont révélés être étroitement liés à la reproduction masculine. Deux des quatre mimotopes (#1 et #25) ont été synthétisés, et un test ELISA a été généré en utilisant ces deux mimotopes comme antigènes spécifiques des spermatozoïdes. Cent trente-quatre échantillons de sérum et soixante-quatorze échantillons de plasma séminal de patients de couples infertiles ont alors été analysés avec ce test ELISA. Respectivement, les échantillons sériques se sont révélés positifs à 20,15% (27/134) pour le mimotope #1 et à 11,19% (15/134) pour le mimotope #25, avec un taux de coïncidence de 91,04% (122/134). Seul un échantillon de plasma séminal (1/74, soit 1, 35%) s’est révélé positif à la fois pour le mimotope #1 et #25 (coïncidence 100%).

**Conclusion:**

La technique « phage display» nous a permis d’identifier des mimotopes d’antigènes spermatiques qui ont pu être utilisés afin de générer un test ELISA pour la détection d’anticorps anti-spermatozoïdes.

## Background

The effects of antisperm antibodies (AsAb) on fertility are well known. Although the WHO Manual recommends the immunobead test (IBT) and mixed antiglobulin reaction (MAR) test for the detection of AsAb [1], the two methods can only detect a single sample and the AsAb on the surface of motile sperm. Moreover, because the two methods need sperm with good motility from fertile men, they are difficult to be routinely used for the detection of AsAb in body fluids such as serum, seminal plasma and cervical mucus in clinics, especially in medical institutions with many patients. In China, the enzyme-linked immunosorbent assay (ELISA) is the most commonly used method for the detection of AsAb [2]. Due to the different preparation methods of sperm antigen used in different ELISA kits for the detection of AsAb, there are differences in the detection results [3]. How to establish a standardized ELISA for the detection of AsAb depends on the standardization of the preparation of sperm antigen coated on the ELISA plate. At present, dozens of sperm antigens have been identified to be associated with infertility, but it is difficult to identify a dominant antigen interacting with all AsAb [3]. Moreover, as a protein, sperm antigen may have cross reaction with antibodies against other proteins in human body. How to avoid the cross reaction of sperm antigens and provide a variety of sperm specific antigens? The combination of several sperm antigen mimotopes as coating antigens may be a better choice at present.

A large number of studies have shown that the phage display technique is a better technology to obtain multiple specific antigen epitopes. Since Smith et al. [4] established the phage display technique in 1985, it has been applied in many fields. The ligand-receptor interactions forming the basis of phage display have been well utilized in epitope mapping and antigen presentation on the surface of bacteriophages for screening novel vaccine candidates [5]. This versatile technique has been modified tremendously over last three decades, leading to the generation of different platforms for combinatorial peptide display, such as phage libraries displaying specific proteins [6], nanobody phage display libraries [7], pan-coronavirus phage display libraries [8], phage-displayed single-chain fragment variable (scFv) libraries [9], etc. The so-called phage display technique is to make foreign DNA fragments inserted into the phage genes encoding coating proteins PIII or PVIII so that a large number of fusion proteins combined random peptides with the coating proteins of filamentous phage are displayed on the surface of bacteriophages. As a large number of nucleotide fragments may be cloned into the phage genome, a phage library may harbor millions or sometimes billions of unique and distinctive displayed peptide ligands [5]. If the peptide ligands interacting with specific antibodies can be screened out from the phage display library by monoclonal antibodies or polyclonal sera, they can simulate continuous or discontinuous epitopes of specified proteins. In this way, meaningful specific antigen epitopes can be obtained when specific antigens are unknown.

At present, studies on antigen epitopes by the phage display technique have been reported in many fields. For example, the epitopes and/or mimotopes for severe acute respiratory syndrome coronavirus 2 (SARS-CoV-2) [8, 10, 11], *Mycoplasma pneumoniae* [12], hepatitis C virus [13], human immunodeficiency virus [13], major capsid protein L1 of human papillomavirus types 18 and 45 (HPV18/45) [14], bovine viral diarrhea virus (BVDV) [15], influenza virus H1N1 [16], RhD antigen [17], influenza virus [18], norovirus [19] and etc. have been obtained successfully by using specific antibodies to screen phage display library combined with gene sequencing. These studies demonstrated that the obtained epitopes/mimotopes could be linear or conformational [11, 18, 20], and that these epitopes/mimotopes were feasible for serological diagnosis and vaccine [10], and were more specific and sensitive [21, 22]. However, there were few reports on the use of the phage display technique in the field of infertility. Williams et al. [23] demonstrated that the sera of infertile women could react with the peptide fragments of human fertilization antigen 1 (FA-1) and YLP12 and suggested that these peptides might be used for the specific diagnosis and treatment of female infertility and the development of contraceptive vaccines. Naz [24] also confirmed that the combination of multiple sperm antigen epitopes could be used as an effective contraceptive vaccine. Samoylova et al. [25] screened the phage display library with zona pellucida (ZP) and also obtained some effective sperm antigen epitopes as contraceptive vaccines. Chen et al. [26] screened the phage display library by the serum of infertile women with positive AsAb, and the obtained four epitopes also had an immunocontraceptive effect. Baldeon-Vaca et al. [27] found that the glycogen epitope of CD52g rich in sperm was also a female contraceptive candidate. Mortazavi et al. [28] immunized mice with multiple sperm antigen epitopes, leading to the infertility of male mice. The sperm epitopes obtained in these studies exerted a contraceptive effect by stimulating the body to produce corresponding antibodies. Similarly, it was also feasible to use these epitopes as antigens to detect AsAb. Therefore, in this study, we used serum with positive AsAb to screen a phage display peptide library, and some meaningful sperm antigen epitopes/mimotopes were obtained, which laid a foundation for the establishment of an ELISA for the detection of AsAb based on sperm antigen epitopes/mimotopes.

## Materials and methods

### Reagents and instruments

The Ph.D.-12 phage display peptide library containing 1 × 10^13^ pfu/ml and *Escherichia coli* ER2738 were purchased from New England Biolabs Inc. (Beverly, MA, USA). Horseradish peroxidase (HRP)-conjugated anti-M13 phage monoclonal antibody was purchased from GE Healthcare (Pittsburgh, PA, USA). HRP-conjugated sheep anti-rabbit IgG was purchased from Sino-American Biotechnology Co., Ltd. (Beijing, China). Taq DNA polymerase and polymerase chain reaction (PCR) amplification kits were purchased from Takara Bio Inc. (Tokyo, Japan). The routine ELISA kit for the detection of AsAb and the negative control, positive control, enzyme conjugate, substrate, and termination solution used for the ELISA method established with synthetic peptides were purchased from Nanjing Xindi Biopharmaceutical Co., Ltd. (Nanjing, China). The Model 550 microplate reader was purchased from Bio-Rad Laboratories (Hercules, CA, USA). The DNA thermal cycler 9600 was purchased from PE Applied Biosystems (Foster City, CA, USA). The super-clean workbench (SW-CJ-1D) was purchased from Suzhou Purification Equipment Co., Ltd. (Suzhou, China). The high-speed refrigerated centrifuge (TLL-C) and constant-temperature shaker (HX-10555) were obtained from Beijing Sihuan Scientific Instrument Factory (Beijing, China).

### Human samples

Normal semen samples were collected from thirty fertile men with normal semen parameters according to the WHO manual [29], and their wives were demonstrated to be infertile mainly due to fallopian tube factors. They asked for *in vitro* fertilization-embryo transplantation (IVF-ET) at our center to help them get pregnant. Spermatozoa from five fertile men each time were mixed to immunize rabbits for a total of six times. In addition, 134 serum samples and 74 semen samples were collected from infertile couples who performed assisted reproductive technology in our hospital and were used for the detection of AsAb. Serum samples came from 69 women and 65 men, aged 33.38 ± 6.96 (24 ~ 51) years and 33.91 ± 6.93 (25 ~ 52) years, respectively. Semen characteristics of 30 fertile men and 74 infertile men were shown in Table 1. All samples were the remaining samples after routine testing. Liquefied semen samples were centrifuged at 3000 g for 15 min to separate sperm and seminal plasma for standby.

**Table 1:** Semen characteristics of 30 fertile men and 74 infertile men (mean ± standard deviation, range)

Group	*n*	Age (years old)	Abstinence time (day)	Semen volume (ml)	Sperm concentration (× 10^6^/ml)	Progressive motility (%)	Sperm motility (%)	Normal morphological sperm (%)
Fertile men	30	31.60 ± 4.12 (25 ~ 43)	3.87 ± 1.36 (2 ~ 7)	3.11 ± 1.13 (1.5 ~ 6)	90.37 ± 62.81 (19.5 ~ 336.8)	43.98 ± 6.63 (34.2 ~ 59.7)	66.17 ± 11.02 (44.5 ~ 92.7)	5.20 ± 0.81 (4.0 ~ 6.5)
Infertile men	74	31.80 ± 5.43 (21 ~ 48)	3.59 ± 1.43 (2 ~ 7)	3.26 ± 1.35 (1 ~ 7)	65.82 ± 43.11 (6.2 ~ 207.1)	26.53 ± 11.48 (3.7 ~ 64.9)	43.59 ± 14.25 (9.1 ~ 79.3)	2.16 ± 1.24 (0.5 ~ 5)

Semen samples from fertile men were used to immunize rabbits, and semen samples from infertile men were used to detect anti-sperm antibodies

### Experimental animals

Four New Zealand male rabbits (No. qls04-0202), weighed 3–5 kg, were purchased from Qinglongshan Animal Breeding Center (Nanjing, China). Among them, two were immunized with human sperm, and two were used for the control. The study was approved by the Institutional Animal Care and Use Committee of Southeast University (No. 20200402030).

### Preparation of rabbit sera with positive AsAb

Spermatozoa from normal fertile men were washed with normal saline, and then sperm concentration was adjusted to about 4 × 10^8^/ml. Two New Zealand male rabbits were immunized with 1 ml of sperm suspension every ten days for six times by the intradermal and subcutaneous multi-point injection. The other two rabbits were immunized with normal saline by the same injection way. One week after the last immunization, all of the blood was collected, and sera were separated and stored at -20℃ for standby.

### Extraction and purification of serum immunoglobulins

Immunoglobulins (Igs) in the rabbit sera with positive AsAb and negative AsAb were extracted by the salting out method, respectively [30]. First, 2 ml of serum was mixed with 2 ml of 0.1 mol/L PBS (pH7.4), and then the mixed solution was slowly added into 4 ml of saturated ammonium sulfate solution drop by drop while shaking. After the obtained solution was stayed at 4℃ overnight, it was centrifuged, and the sediment was dissolved with 5 ml of PBS. Next, the 5 ml of solution was slowly added into 2.5 ml of saturated ammonium sulfate solution drop by drop while shaking, and the obtained solution was stayed at 4℃ overnight. Repeat the last operation (centrifugation, dissolution, and precipitation) once. Last, the precipitate was dissolved with 4 ml of PBS and dialyzed against normal saline. The antibody titer and protein concentration of the final solution were detected by ELISA and an automatic biochemical analyzer, respectively.

### Screening of phages with sperm antigen epitopes

The following biopanning, adsorption for removing nonspecific phages and determination of phage titers were carried out in accordance with the operating instructions of the Ph.D.-12 phage display peptide library kit.

#### Biopanning

The wells of a microtitre plate were coated overnight at 4℃ with 100 μl of purified AsAb-Igs (100 μg/ml in carbonate buffer, pH 8.5) in a humidified container. Blocking buffer (15% bovine serum albumin [BSA] in 0.1 mol/L phosphate buffer saline [PBS]) was then added for 2 h at 4 °C to block the wells. After washing six times with TBST (50 mmol/L Tris–HCl, 150 mmol/L NaCl, 0.1% Tween-20, pH 7.5), the wells were incubated for 1 h at 37 °C with 1 μl of library phages (~ 1 × 10^10^ pfu) diluted in 100 μl of TBST. The wells were next washed ten times with TBST to remove any unbound phages. The bound phages were eluted with 100 μl of elution solution (1 mg/ml BSA in 0.2 mol/L glycines, pH 2.2) for 10 min at room temperature and neutralized with 15 μl of 1 mol/L Tris–HCl (pH 9.1).

#### Adsorption for removing nonspecific phages

The above-eluted phages were added into the wells of a microtitre plate coated with AsAb-negative rabbit serum Igs for 1 h at room temperature and the operation was repeated for six times to remove nonspecific phages.

#### Determination of phage titers

The adsorbed phages were diluted with liquid LB culture medium (5 g/L NaCl, 5 g/L yeast extract, 10 g/L tryptone, 15 g/L agar) at 1:10, 1:100, 1:1000 and 1:10,000 times, and then 10 μl of dilution for each titer were mixed with 200 μl of *Escherichia coli* ER2738 cultured overnight. After incubation for 5 min, the mixture was quickly mixed with 3 ml of preheated top agar (10 g/L tryptone, 5 g/L yeast extract, 5 g/L NaCl, 1 g/L MgCl_2_ • 6H_2_O, 7 g/L agar), and immediately added to the LB plate preheated at 37℃. The LB plate contained X-gal and isopropyl-beta-D-thiogalactoside (IPTG) for screening the blue bacterial colonies with bacteriophages. After being cultured overnight at 37℃, the blue colonies on the LB plate were counted, and the phage titers were calculated.

### Amplification of positive phage clones

Both of 1 μl of *Escherichia coli* ER2738 cultured overnight and one positive clone (one blue bacterial colony) were added into 1.5 ml of LB culture medium and cultured in a shake flask for 5–6 h. After centrifuging for 15 min at 12 000 r/min, the supernatant containing the required phages was collected, and its phage titer was determined.

### Further screening of positive phage clones by ELISA

Modified indirect ELISA and sandwich ELISA were further used to screen positive phage clones. The specific operations were as follows.

#### Indirect ELISA

The wells of an ELISA plate were coated with 10^8^ pfu of positive phages overnight at 4℃ and blocked with 15% BSA in 0.1 mol/L PBS (pH 7.4) for 2 h at 4℃. The rabbit AsAb-Igs diluted at 1:500 times were added to the phages-coated wells and incubated for 30 min at 37℃. PBS was used as the negative control. After washing five times with PBST (0.1% Tween-20 in 0.1 mol/L PBS), 1:1000-diluted HRP-conjugated sheep anti-rabbit IgG in blocking buffer (15% BSA in 0.1 mol/L PBS, pH 7.4) was added to the wells and incubated for 30 min at 37℃. The wells were rewashed and peroxidase activity was detected by adding 3,3',5,5'-tetramethylbenzidine (TMB) substrate. The optical density (OD) at 450 nm was subsequently recorded using a microplate reader. If the OD value was more than 2.1 times of the negative control, the positive phage clone was picked out.

#### Sandwich ELISA

The wells of an ELISA plate were coated with purified AsAb-Igs overnight at 4℃ and blocked with 15% BSA in 0.1 mol/L PBS (pH 7.4) for 2 h at 4℃. Phage clones (10^8^ pfu) were added to the AsAb-Igs-coated wells and incubated for 2 h at room temperature with agitation. TBS (50 mmol/L Tris–HCl, 150 mmol/L NaCl, pH 7.5) was used as the negative control. After washing five times with PBST, 1:2500-diluted HRP-conjugated anti-M13 antibody in blocking buffer was added to the wells and incubated for 1 h at room temperature with agitation. The wells were rewashed and peroxidase activity was detected by adding TMB substrate. The OD at 450 nm was subsequently recorded using a microplate reader. If the OD value was more than 2.1 times of the negative control, the positive phage clone was picked out.

### PCR amplification and sequencing of positive phage clones

The phage single-strand DNAs (ssDNA) of positive phage clones detected by both indirect ELISA and sandwich ELISA were extracted according to the instructions of the kit. Then, the phage ssDNA was amplified with the specific forward primer (5ˊ- CGCAATTCCTTTAGTGGTAC-3ˊ) and reverse primer (5ˊ- CCCTCATAGTTAGCGTAACG-3ˊ) designed according to the phage DNA sequence. The PCR amplification conditions were as follows: preheating to 94℃ for 5 min; followed by 35 cycles of denaturation at 94℃ for 30 s, annealing at 55℃ for 30 s, and elongation at 72℃ for 30 s; and with a final elongation step at 72℃ for 7 min. Reaction products (~ 200 bp) were assessed by electrophoresis on 2% agarose. PCR products were collected and sent to the TaKaRa Bio Inc. for sequencing.

### Synthesis of peptides

The DNA sequences obtained were translated into amino acid sequences and subjected to Blast (https://blast.ncbi.nlm.nih.gov/Blast.cgi) in a non-redundant protein database to detect mimotopes matching sperm membrane antigens. Furthermore, the peptides #1 (VDTVWTVKMGRV) and #25 (DGDPRGPDSHQI) were synthesized by the Beijing Huada Genetic Research Center (Beijing, China) for further study. Their purities were 96.73% and 95.32%, respectively, and concentrations were 10 mg/ml.

### Establishment of the ELISA method for detecting AsAb in serum and seminal plasma samples

The ELISA method was established based on the modification of routine ELISA. The specific operations were as follows. The polypeptide was diluted at 1:200 with carbonate buffer (pH9.6) and coated onto the well of a polystyrene plate with 100 μl (500 μg) per well. After overnight at 4℃, the coating solution was poured out, and each well was filled with blocking buffer (15% BSA in 0.1 mol/L PBS, pH7.4) for 2 h at 4℃. The wells were washed with PBST for five times, and then 100 μl of sample diluent and 5 μl of serum sample, or 50 μl of sample diluent and 50 μl of seminal plasma sample, were added into each well. Meanwhile, negative and positive controls were set, and 100 μl of negative control and positive control serum or seminal plasma were added into a well. The positive samples screened from clinical samples were selected as the positive control, and the serum or seminal plasma samples from fertile men as the negative control. After incubating for 40 min at 37℃, the wells were washed with PBST for five times, and 100 μl of HRP-conjugated sheep anti-human IgG was added into each well. After incubating for 30 min at 37℃, the wells were washed with PBST for five times, and peroxidase activity was detected by adding 3,3',5,5'-tetramethylbenzidine (TMB) substrate. The OD at 450 nm was subsequently recorded using a microplate reader. If the OD value was more than 2.1 times of the negative control, the sample was judged as positive.

### Statistical analysis

The negative and positive results of AsAb in 134 serum samples and 74 seminal plasma samples detected with #1 and #25 polypeptides as coating antigens and the routine ELISA kit were input into the Excel table, and the positive rates of each detection method and the coincidence rates between different methods were obtained by a simple calculation. The comparisons of positive rates of AsAb between male and female serum samples were analyzed by the χ^2^ test of the SPSS 22.0 statistical software (SPSS Inc., Chicago, IL, USA), and *P* ≤ 0.05 was considered to be statistically significant.

## Results

### Titers of AsAb-IgG

After immunoglobulins in serum with positive AsAb were extracted and purified by the saturated ammonium sulfate method, further dialyzed and concentrated, the protein concentration of the obtained solution was 50.6 mg/ml. The titer of AsAb-IgG detected by ELISA was more than 1:320.

### Screening results of peptide library

**Fig. 1:**
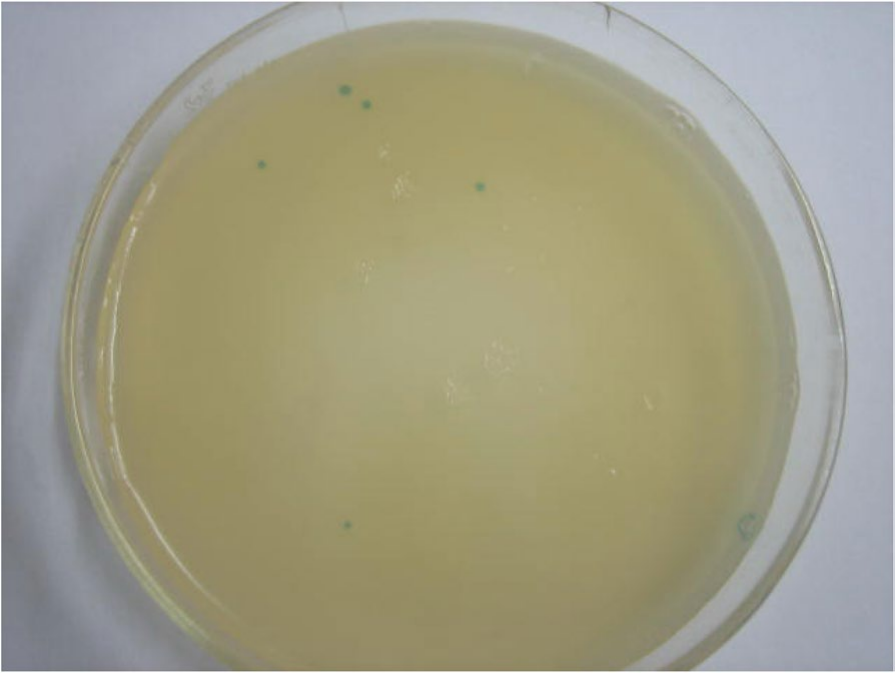
Positive phage clones (blue plaque) on an LB/X-gal/IPTG plate. The Ph.D.-12 phage display peptide library was panned by the microtitre plate coated with AsAb-IgG, and the eluted phages were further adsorbed with AsAb negative-IgG. Then, the adsorbed phages were mixed with *Escherichia coli* ER2738 and cultured overnight. The culture mixture was quickly mixed with preheated top agar, and immediately added onto the LB plate containing X-gal and isopropyl-beta-D-thiogalactoside (IPTG) for screening the blue bacterial colonies with bacteriophages. The blue dots in the figure were positive phage clones

After the Ph.D.-12 phage display peptide library was panned by the microtitre plate coated with AsAb-IgG, the eluted phages were further adsorbed with AsAb negative-IgG, and a total of sixty positive phage clones (blue plaque) were obtained by the determination of phage titers (Fig. 1).

### Identification of positive phage clones by ELISA

After sixty positive phage clones were amplified by culture, they were further identified by indirect ELISA and sandwich ELISA. A total of sixteen clones, which were positive in both indirect ELISA and sandwich ELISA, were obtained, and their numbers were as follows: #1, #4, #5, #16, #18, #25, #27, #29, #32, #34, #35, #54, #56, #57, #58 and #59.

### PCR amplification and analysis of sequencing results

**Fig. 2:**
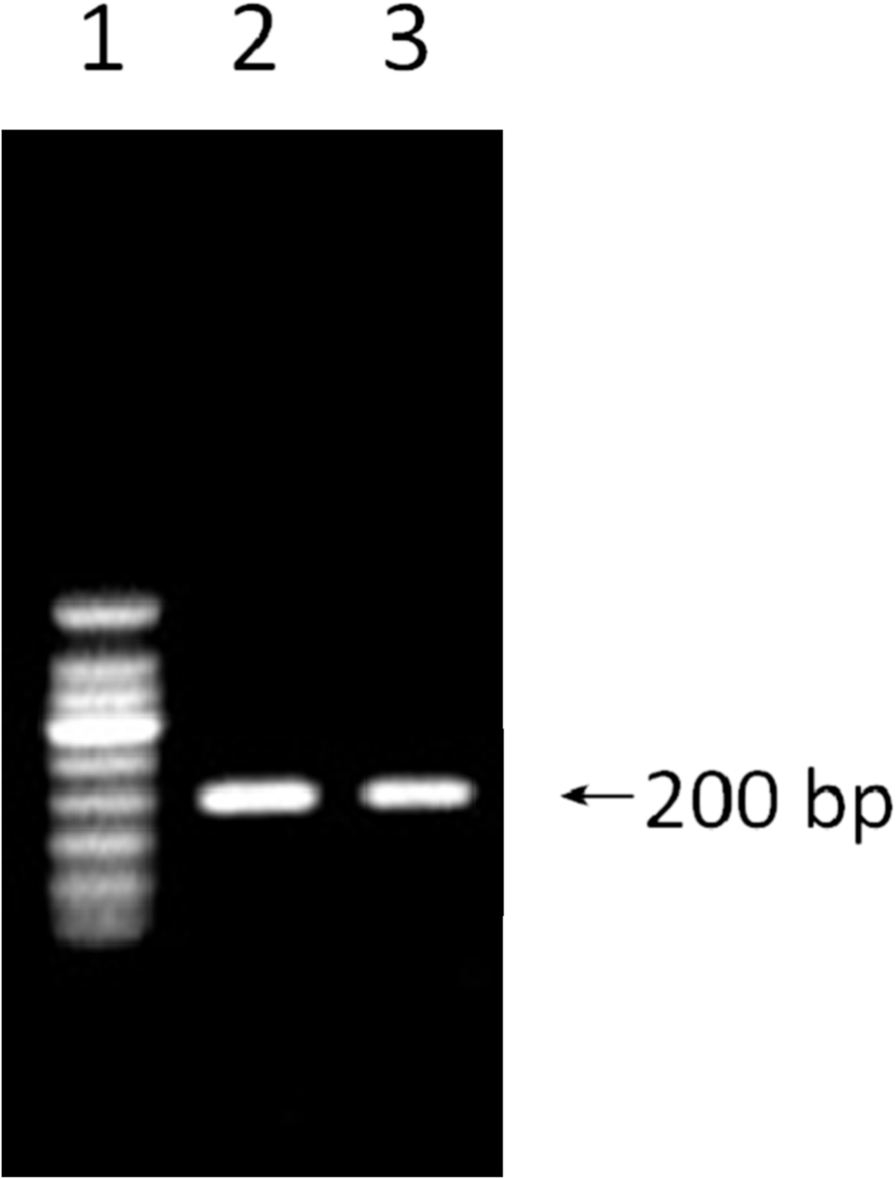
PCR amplification results of positive phage DNA. 1: DNA marker; 2, 3: PCR products. The DNA of positive phage clones was extracted and amplified by PCR, and PCR products (~ 200 bp) were assessed by electrophoresis on 2% agarose

**Fig. 3:**
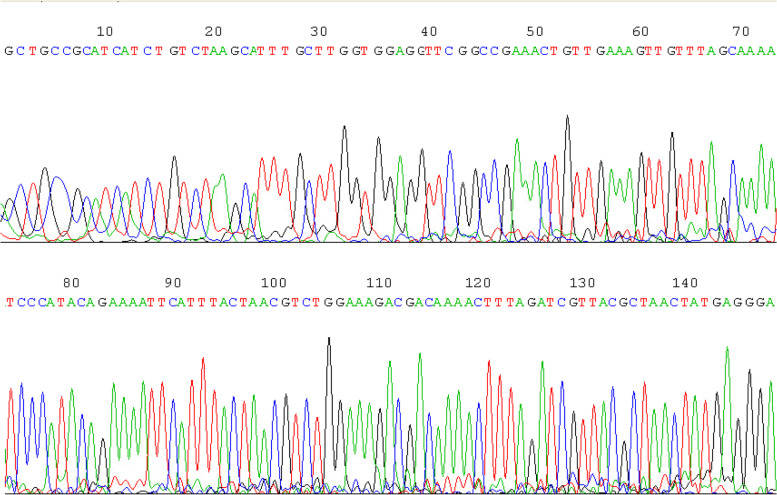
Sequencing results of PCR products. The DNA of positive phage clones was extracted and amplified by PCR, and PCR products were sent to TaKaRa Bio. Inc. for sequencing. Here is the sequencing result of #1 clone

The DNA of sixteen positive phage clones was extracted and amplified by PCR, and the results are shown in Fig. 2. PCR products were sent to TaKaRa Bio. Inc. for sequencing, and the results were compared with the DNA sequence of the M13 phage. The DNA sequences obtained (Fig. 3) were translated into amino acid sequences and subjected to Blast (http://blast.ncbi.nlm.nih.gov/Blast) in a non-redundant protein database to analyze their homology, and the results are shown in Table 2. Among these homologous sequences, the sequences of #1, #16, #25, and #54 clones had high homology with male reproductive system related proteins. Five amino acids (4–8) of #1 clone were identical to that of sperm protein SSP411 (418–422). The peptide sequence of #16 clone was similar to that of KIF27C. The peptide sequence of #25 clone was homologous with those of SCRL and THEG proteins. Six amino acids of #54 clone were the same as those of epididymal specific lipocalin-10 and putative doublecortin domain-containing protein.

**Table 2:** BLAST analysis results of amino acid sequences of positive phage clones

Clone (No.)	Sequence	Homology
1	VDTVWTVKMGRV	FAM82C protein (6/12); cerebral protein-10 (6/12); NDUFA6 (6/12); sperm protein SSP411 and Spermatogenesis associated 20 (5/12)
4	EHPRLVGNPVKS	additional sex combs like 2 isoform 4 (10/12); selectin-like protein (6/12); polydom isoform 6 (6/12); RP11-427L11.3 (6/12)
5	DGHLLTSRFLLH	FLJ00025 protein (6/12); Intraflagellar transport 122 homolog (Chlamydomonas) (6/12); WDR10p (6/12); ZNF287 protein (6/12)
16	LGHVIFSTSIVH	hypothetical protein LOC9703 (8/12); KIF27C (7/12); intestinal mucin (6/12); MUC3B mucin (6/12)
18	TVPKETTPLTLT	tRNA selenocysteine associated protein (7/12); chromosome 6 open reading frame 52 (7/12); neural cell adhesion protein (7/12); C6orf52 protein (7/12)
25	DGDPRGPDSHQI	SCRL protein (7/12); THEG protein (6/12); Theg homolog isoform 2 (6/12); Granzyme A (6/12)
27	STQPAPVTIEQG	KIAA1324 protein (8/12); LIM domain only 4 (6/12); DNA-binding protein B (7/12); expressed in hematopoietic cells, heart, liver (6/12)
29	CNMTYKIYALHD	zinc finger, FYVE domain containing 26 (7/12); Leukocyte common antigen precursor (6/12); protein tyrosine phosphatase(6/12); T200 leukocyte common antigen precursor (6/12)
32	VPIKLNLYRSVH	chromosome 16 open reading frame 7 (7/12); ankyrin repeat-containing protein (6/12); nebulin (6/12); ZFP28 protein (7/12)
34	RILATPVSALAV	Bile acid Coenzyme A: amino acid N-acyltransferase (glycine N-choloyltransferase) (7/12); BAAT (7/12); hormonally upregulated Neu-associated kinase (8/12); Trinucleotide repeat containing 4 (7/12)
35	ARAPSSSAFSLL	Ig heavy chain variable region (8/12); circulating B cell antibody heavy chain variable region (8/12); UDP-glucuronosyltransferase (10/12); inner centromere protein (8/12)
54	ATVPPVGQVRVL	ZNF574 protein (7/12); putative doublecortin domain-containing protein (6/12); human immunodeficiency virus type I enhancer binding protein (7/12); Epididymal-specific lipocalin-10 precursor (6/12)
56	WQLPHHLSKHLL	ZNF206 protein (7/12); zinc finger protein 237 (8/12); Signal peptide peptidase-like 2B (7/12); PES1 (6/12)
57	SVADKLALVRPP	histone deacetylase 10 (7/12); OTTHUMP00000042163 (7/12); ZNF672 protein (8/12); Monocarboxylate transporter 7 (6/12)
58	YTMQSSGISRWQ	choroideremia-like protein (6/12); OATP-I (6/12); zinc finger protein 639 (6/12); retinoblastoma-associated factor 600 (6/12)
59	SSYTPIQMPYNT	tRNA isopentenyl transferase (7/12); Chromosome 10 open reading frame 130 (6/12); metallophosphoesterase domain containing 1 (6/12); OTTHUMP00000028881 (6/12)

The DNA of sixteen positive phage clones was extracted and amplified by PCR, and PCR products were performed sequencing. The DNA sequences obtained were translated into amino acid sequences and subjected to Blast (http://blast.ncbi.nlm.nih.gov/Blast) in a non-redundant protein database to analyze their homology. Among these homologous sequences, the sequences of #1, #16, #25, and #54 clones had high homology with male reproductive system related proteins. Five amino acids (4–8) of #1 clone were identical to that of sperm protein SSP411 (418–422). The peptide sequence of #16 clone was similar to that of KIF27C. The peptide sequence of #25 clone was homologous with those of SCRL and THEG proteins. Six amino acids of #54 clone were the same as those of epididymal specific lipocalin-10 and putative doublecortin domain-containing protein

### Establishment of ELISA based on sperm antigen epitopes and analysis of detection results

The ELISA method of AsAb detection was established with #1 and #25 polypeptides as coating antigens, respectively. When 134 serum samples were detected by the established ELISA methods using #1 and #25 polypeptides as sperm-specific antigen epitopes, respectively, the positive rates of AsAb were 11.19% (15/134) and 20.15% (27/134), respectively. The positive rate of AsAb detected by the routine ELISA kit was 11.94% (16/134). When 74 seminal plasma samples were detected by the three ELISA methods, the positive rates of AsAb for them were 1.35% (1/74).

Although the positive rate of AsAb detected with #25 polypeptide as antigen epitope was higher than that of #1, the coincidence rate between them was 91.04% (122/134), and the 12 inconsistent samples with positive AsAb were from #25 polypeptide as antigen epitope. The coincidence rate between the ELISA method using #25 polypeptide as antigen epitope and the routine ELISA kit was 88.81%. It was suggested that #25 polypeptide as coating antigen should be more sensitive than #1 and conventional sperm membrane antigens and that #1 polypeptide as coating antigen be more specific.

In addition, the positive rates of AsAb from male and female serum samples detected with #1 polypeptide as antigen epitope were 9.23% (6/65) and 13.04% (9/69), respectively, and those detected with #25 polypeptide as antigen epitope were 16.92% (11/65) and 23.19% (16/69), respectively. The positive rates of AsAb detected by the routine ELISA kit for male and female serum samples were 9.23% (6/65) and 14.49% (10/69), respectively. The positive rates of AsAb from male serum samples were lower than that from female serum samples for the three ELISA methods with different coating antigens, but there was no statistical difference between them (*P* > 0.05).

### Discussion

The causes of infertility are very complex. Immunological factors are closely related to the pathogenesis of some infertile patients. Under normal circumstances, the blood-testis barrier and the epididymal blood-epithelium barrier in humans are important structures in preventing sperm antigens from contacting immunocompetent cells, due to the tight junctions of Sertoli cells and epithelial cells. The immunosuppressive substances in semen and follicular fluid and suppressor T lymphocytes in the human immune system, which partially mediate the normal state of immunologic unresponsiveness toward sperm autoantigens, also play an important role in preventing the autoimmune response. In addition, polymorphonuclear neutrophils in semen and high concentrations of IgG and IgA in the uterine fluid could eliminate nonviable sperm or debris [3]. If these protective mechanisms were disrupted, an autoimmune response against human sperm would occur, which may lead to infertility in men and women. It was reported that vasectomy, vasovasostomy, focal cryptic obstruction, testicular torsion, biopsy and cancer, infection and intercourse during the menstrual period could lead to immunogenic sperm antigens being exposed to the immune system thus initiating an immune response to produce AsAb [3]. AsAb can negatively affect sperm motility, cervical mucus penetration, capacitation, acrosome reaction (AR), zona pellucida (ZP) recognition and the fusion of gametes, by immobilizing and agglutinating sperm, and blocking sperm-egg interaction. AsAb can also lead to abortion by preventing implantation, and/or arresting embryo development. It is generally believed that the presence of AsAb may be related to low fertility but not absolute infertility [3].

Different incidences of AsAb reported in the infertile population may be due to different detection methods. Immunobead test (IBT) and mixed antiglobulin reaction (MAR) methods are elementary, inexpensive, and rapid, but they need to count at least 200 motile sperm which carry or do not carry immunobeads. The two methods are subjective due to observation under a microscope. Moreover, the positive rates of AsAb detected by them are very low (from our clinical practice), which may be related to the fact that the two methods require the binding of antigen and antibody to be completed within three minutes, and that the immunobeads should be firmly bound to motile sperm within three minutes. In addition, when indirect IBT and MAR methods are used to detect AsAb in body fluids such as seminal plasma, serum, and cervical mucus, they need fresh sperm from normal fertile men, which is extremely difficult in clinics. Furtherly, the two methods can only detect one sample at a time, which is unsuitable for the medical institutions with a lot of infertile patients. Therefore, it is necessary to establish an ELISA method for rapidly screening AsAb, and the positive ones can be further verified by the MAR or IBT method.

Compared with the IBT and MAR methods, an ELISA test is more sensitive and less subjective, does not need fresh sperm, and can detect a large number of samples at a time and perform an epidemiological investigation on the incidence of AsAb. The variations of the results on the detection of AsAb among different brands of ELISA kits mainly come from the different preparation procedures for sperm antigens. It was reported that sperm antigens using an ELISA test were prepared by different methods, such as lithium 3,5-diiodosalicylate (LIS), nonidet P-40 (NP-40), dithiothreitol (DTT), 3-[(3-choleamidopropyl) dimethylammonio]-1-propane sulfonate (CHAPS), sonication, cavitation and gene engineering [3]. Due to the complexity of the composition of sperm antigens, sperm membrane antigens prepared using different methods could produce widely varying results for different brands of ELISA kits. To establish a standardized method for the preparation of sperm antigens and a standardized and universally accepted ELISA test for the detection of AsAb, we obtained several specific sperm antigen mimotopes by phage display technique. A similar ELISA test based on the mimotopes had been used for the diagnosis of pulmonary tuberculosis [31].

In this study, we first immunized rabbits using human sperm to obtain sera with positive AsAb. After immunoglobulins in the sera were extracted and purified by the salting out method, the titer of AsAb-IgG was more than 1:320, which could be used to screen the phage display peptide library. After several rounds of screening and verification, sixteen positive phage clones were obtained, indicating that these positive phage clones carried mimotopes which could specifically bind to AsAb. To ensure that these mimotopes were related to human sperm as much as possible, the homology of sixteen positive phage clones was analyzed by the Blast web, and it was found that the polypeptide sequences of #1, #16, #25, and #54 clones were highly homologous with those of male reproductive system related proteins. Sperm protein SSP411 homologized with #1 clone was a member of the sperm-specific thioredoxin family and played a role in sperm maturation, fertilization and embryonic development [32]. KIF27C protein [33], which was homologous with #16 clone, was mainly expressed in testicular cells and played an important role in their growth and function. The same amino acids between #16 clone and KIF27C were not continuous, suggesting that the polypeptide sequence of #16 clone might simulate the conformational epitope of KIF27C protein. SCRL protein homologized with #25 clone was highly expressed in testis and prostate and THEG protein homologized with #25 clone was expressed in sperm cells, which were related to male reproduction [34]. Epididymal specific lipocalin-10, which was homologous with #54 clone, played an important role in male reproduction, while putative doublecortin domain-containing protein was mainly expressed in testis.

Subsequently, two polypeptides from #1 and #25 clones, which were highly homologous with male reproductive system related proteins, were synthesized in vitro and used as coating antigens for establishing an ELISA method. Clinical serum and seminal plasma samples were detected by the established ELISA method, and it was found that their results were highly consistent with those of routine ELISA. The consistency of serum samples was more than 88%, and that of seminal plasma samples was 100%. Moreover, the background of the established ELISA method using polypeptides as coating antigens was significantly lower than that of routine ELISA. It was suggested that the established ELISA method based on sperm antigen epitopes could be routinely used to detect AsAb, and that the judgment of the results was more precise and accurate. In addition, when the established ELISA method was used to detect AsAb raised in rabbits, strong positive results were obtained, which verified the feasibility of the phage display technique in screening sperm antigen epitopes again. Therefore, using the mixture of several sperm antigen epitopes as coating antigens in the future may not only improve the detection sensitivity of AsAb, but also increase the detection specificity [35, 36]. Moreover, the use of mixed mimotopes may help avoiding the risk of biological harm and establish a standardized detection method of AsAb [37].

## Conclusions

In order to establish a standardized ELISA method for detecting AsAb, we obtained AsAb-IgG by immunizing rabbits with normal fertile male sperm, and sixteen positive phage clones by biopanning the phage display library. After analysis on the Blast web, four sperm antigen mimotopes closely related to male reproduction were acquired, and two of them were synthesized *in vitro* to establish an ELISA method. AsAb of 134 serum samples and 74 seminal plasma samples were detected by the established ELISA and routine ELISA, respectively, and there was good consistency between them, indicating that sperm antigen mimotopes could be used as standard sperm-specific antigens for establishing an ELISA method for the detection of AsAb.

## Data Availability

The datasets used and/or analyzed during the current study are available from the corresponding author on reasonable request.

## Abbreviations

AR: Acrosome reaction
AsAb: Antisperm antibody
BSA: Bovine serum albumin
BVDV: Bovine viral diarrhea virus
CHAPS: 3-[(3-Choleamidopropyl) dimethylammonio]-1-propane sulfonate
DTT: Dithiothreitol
ELISA: Enzyme-linked immunosorbent assay
FA-1: Fertilization antigen 1
HPV: Human papillomavirus
HRP: Horseradish peroxidase
IBT: Immunobead test
Igs: Immunoglobulins
IPTG: Isopropyl-beta-D-thiogalactoside
IVF-ET: *in vitro* Fertilization-embryo transplantation
LIS: Lithium 3,5-diiodosalicylate
MAR: Mixed antiglobulin reaction
NP-40: Nonidet P-40
OD: Optical density
PBS: Phosphate buffer saline
PCR: Polymerase chain reaction
SARS-CoV-2: Severe acute respiratory syndrome coronavirus 2
scFv: Single-chain fragment variable
ssDNA: Single-strand DNA
TMB: 3,3',5,5'-Tetramethylbenzidine
ZP: Zona pellucida
